# New Appliances and Things Medical

**Published:** 1908-08-22

**Authors:** 


### NEW APPLIANCES & THINGS MEDICAL.

### "CHLOROPHYLLE" TINTED GLASSES.

The ideal coloured glass for the protection of the eyes from strong sunlight, whether direct or reflected, has been the object of much investigation. The familiar smoke-coloured and bottle-green spectacles have been used so widely and so long, not because they gave full satisfaction but principally because nothing much better has been available. Since 1880 the tinted glasses introduced by Dr. Fieuzal have been well known to ophthalmic surgeons. They were a distinct advance upon the commoner kinds of eye protector, but they did not quite meet all requirements, and their adoption has not been universal. Their manufacture, also, is expensive, which is a considerable drawback. Fieuzal's glasses, however, convinced many observers of the superiority of a greenish-yellow colour over blue, greenish-blue, plain green, or yellow, and even over neutral tints of various kinds. Following upon the lines of Dr. Fieuzal's researches, Dr. Leon Fargier has recently produced a coloured glass which he claims to be perfectly suited to all the requirements of comfort and clear vision. Dr. Fargier, experimenting with various specimens of Fieuzal glass, was struck by the analogy between a particular tinted glass and the yellowish tone imparted to green leaves by bright sunlight passing through them. Reasoning from this, and impressed by the soothing effect upon the eyes of sunshine tempered by transmission through green foliage, he decided to follow Nature and imitate the colour of chlorophyll in his tinted glasses. The result has been the production of a perfected type of Fieuzal yellow-green glass in three definite depths of colour. No. 1 (the lightest) transmits approximately 70 per cent. of light, No. 2, transmits 55 per cent., and No. 3, 30 per cent. We regard these "Chlorophylle" glasses as a distinct advance upon other kinds of eye protector. They abolish the discomfort even of the strongest glare, and are most restful to the eyes. There is surprisingly little loss of clearness of vision, even within doors, while in strong sunshine objects appear with admirable distinctness, and effects of light and shade are slightly intensified. The tint which the glasses impart to things looked at is quite unobjectionable. Towards the violet end of the spectrum the amount of light transmitted is very small—about 1.5 per cent. in the case of No. 3 variety. On theoretical grounds the adoption of the colour of chlorophyll seemed reasonable, and our practical experience of the glasses endorses the claims made on their behalf. For reading and for all out-of-door occupations in summer-time they are most comfortable, and deserve the careful notice of the medical profession. "Chlorophylle" glass is supplied with plane surfaces in all the forms and sizes suitable for motorists' goggles, as well as with plane and curved surfaces for spectacles and eye-glasses. It can be obtained from retail opticians. The manufacturers are Messrs. J. B. Jacquemin Bros., Ltd., 65 Hatton Garden, E.C.

